# Social Networking Versus Facebook Advertising to Recruit Survey Respondents: A Quasi-Experimental Study

**DOI:** 10.2196/resprot.3317

**Published:** 2014-09-17

**Authors:** Conor Gilligan, Kypros Kypri, Jesse Bourke

**Affiliations:** ^1^School of Medicine and Public HealthFaculty of Health and MedicineUniversity of NewcastleCallaghanAustralia; ^2^Hunter Medical Research InstituteNew LambtonAustralia; ^3^Centre for Clinical Epidemiology and BiostatisticsNew LambtonAustralia

**Keywords:** Facebook advertising, recruitment, Facebook

## Abstract

**Background:**

Increasingly, social contact and knowledge of other people’s attitudes and behavior are mediated by online social media such as Facebook. The main research to which this recruitment study pertains investigates the influence of parents on adolescent alcohol consumption. Given the pervasiveness of online social media use, Facebook may be an effective means of recruitment and intervention delivery.

**Objective:**

The objective of the study was to determine the efficacy of study recruitment via social networks versus paid advertising on Facebook.

**Methods:**

We conducted a quasi-experimental sequential trial with response rate as the outcome, and estimates of cost-effectiveness. The target population was parents of 13-17 year old children attending high schools in the Hunter region of New South Wales, Australia. Recruitment occurred via: method (1) social recruitment using Facebook, email-based, social networks, and media coverage followed by method (2) Facebook advertising.

**Results:**

Using a range of online and other social network approaches only: method (1) 74 parents were recruited to complete a survey over eight months, costing AUD58.70 per completed survey. After Facebook advertising: method (2) 204 parents completed the survey over four weeks, costing AUD5.94 per completed survey. Participants were representative of the parents recruited from the region’s schools using standard mail and email.

**Conclusions:**

Facebook advertising is a cost-effective means of recruiting parents, a group difficult to reach by other methods.

## Introduction

### Social Media and Research

Increasingly, online social media and social network sites mediate social contact and perceptions of other people’s attitudes and behavior. There are 728 million people worldwide that use Facebook at least daily [1]. A recent review confirms Facebook’s potential for the study of human behavior [2].

### Facebook Recruitment

Several recent studies report Facebook as a cost effective and expedient method for recruitment [3-7], though not all enjoy success [8]. Successful approaches vary from Facebook network media and advertising alone to advertising through several search engines and websites. While Facebook advertising is widely used for marketing, and is recommended by some researchers [5-7], it is unclear whether this approach is more effective than other online or traditional approaches for securing participation in online surveys or forums. No randomized trials have investigated the effectiveness of recruitment through Facebook relative to other approaches.

### The Current Research

We are interested in investigating the role of parents in influencing adolescents’ alcohol consumption. Engaging parents has historically been a challenge for educators, public health practitioners, and researchers, with low response rates and high attrition plaguing parent- and family-focused interventions [9]. Given the uncommonness of direct connections between parents [10], the pervasiveness of social media use [1], and the success of programs that correct misperceptions about others’ drinking [11-13], Facebook may be a useful means of study recruitment and intervention delivery.

This study investigates the efficacy and cost-effectiveness of method (1) social recruitment using Facebook, email-based, social networks, and media coverage relative to method (2) Facebook advertising to recruit parents to complete an online survey. The outcomes are participant numbers, the representativeness of participants, and the recruitment cost per participant. 

## Methods

### Design

We conducted a quasi-experimental sequential trial of recruitment methods, with method (1) initiated first, followed by method (2) in the same population of parents. Inferences about effects are based on the number and timing of responses resulting from methods (1) and (2). It is not possible to measure the response fraction associated with approach (1), as the number of people who were exposed to our recruitment efforts cannot be determined.

### Participants

The target population was parents in the Hunter Region of New South Wales (population 644,300), Australia. Eligibility was limited to parents of adolescents 13-17 years old attending the 59 secondary schools in the region. The demographic characteristics of participants are summarized in Table 1.

### Procedure

Each of Facebook’s network media was used to create an active parent network and to invite parents to complete the survey. The “Hunter Parents Alcohol Forum” (HPAF) was a closed group designed to pilot the use of this approach for intervention. A HPAF Facebook page was designed to attract participants, and a profile (“Hunter PAF”) was used for forum discussion and to connect with existing groups and pages. All participants who completed the survey or joined the forum were entered into an iPad prize draw.

### Social Network Recruitment and Social Marketing, Method (1)

There were 18 networks likely to include our target population that were identified through Facebook’s search function, and connection was established via “friend request”, “liking”, or “joining”. A private message was sent to the administrator introducing the researchers and the project, and requesting they share the survey and HPAF links among their members. There were three of the networks that posted a description and links to the project on their “wall”, and we indirectly addressed members of other networks through a public posting on their wall.

Invitations to participate and share the links were circulated via email through our own networks of colleagues, friends, and family. The research was featured twice each in a popular regional newspaper and local radio program at morning and evening “drive-times”. An article was featured in a publication with a circulation of 70,000 through regional newspapers. Flyers and recruitment cards were placed in cafes and public places locally, and were disseminated to parents by sporting clubs. There were three clubs that advertised the project in their newsletter. Dr Gilligan spoke at one school’s Parents and Citizens Committee meeting and another school posted information about the study on its website and distributed 1000 flyers to parents in a school-fee mail-out.

Materials distributed as part of this approach included brief notices with Web links and scannable codes, flyers and posters more fully explaining the purpose of the study and what parents were invited to do, and longer written pieces about the background to the larger project and its purpose. The content and images varied for each medium used.

### Facebook Advertising, Method (2)

**Figure 1:**
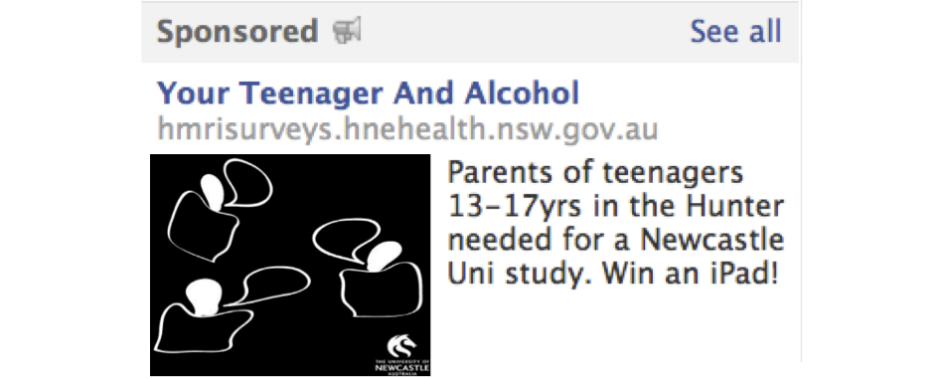
Advertisement used for recruitment as it appeared on potential participants’ Facebook newsfeeds. Text had a 130-character limit.

To generate a Facebook advertisement, clients select from a series of options for the purpose of the advertisement and pricing arrangement (cost per click or per metric), provide text and images, stipulate the Facebook demographic parameters, and specify a budget and time frame. Cost per click is automatically calculated to optimize the number of clicks in accordance with a maximum set by the user. We produced an advertisement (Figure 1 shows this advertisement) targeting people in the Hunter region (based on postcode), age ≥ 30 years, and whose Facebook profiles indicated that they were parents of children 13-15 years or 16-19 years old (the most relevant available parameters). The advertisement ran for five weeks, immediately following from intervention (1), including an initial trial and several modifications to the advertising parameters. The maximum budget was AUD350 per week, and a cost per click arrangement was used. To assess the impact of the “parents of children” variable depending on accurate profile information, we ran one weeklong advertisement without this limitation.

## Results

### Comparative Recruitment Rates and Costs

During the eight month social recruitment phase of the trial, 74 people completed the online survey. The main cost of this approach was in research assistant time (AUD4349 for the eight month period based on 10 hours per week dedicated to recruitment efforts), equating to AUD58.70 per survey completion.

Facebook advertising identified a population of 15,900 individuals meeting our eligibility criteria. In the two weeks of active advertising using parent parameters and the final agreed budget framework, the advertisements generated 259 clicks per week (AUD402 total) and 160 survey completions (AUD2.51 per completion). When the “parents of children variable” was removed in an intervening week, the population size increased to 182,000, but the number of clicks decreased (215 clicks costing AUD344 in total) and survey completions (29 completions at AUD11.90 each) also declined.

In total, AUD1107 was spent on advertising, 840 clicks were generated, and 204 surveys were completed during this period. The cost per completed survey was AUD5.94, including one month of a research assistant wage.

### Study Participants and the Parent Population Comparison

Table 1 presents a comparison (for the purposes of assessing population representativeness) between the study participants and the population of parents recruited from three Hunter schools using more traditional postal and electronic mail techniques in a separate study conducted in the same year (Gilligan et al, 2014 unpublished data).

**Table 1:** Characteristics of parents in this study compared with parents recruited by post and email from Hunter schools in a separate study (assessing sample representativeness).

	Participants in the present study	Hunter schools study
	Total (N=278), mean (SD)	Total (N=444), mean (SD)
Adolescent age	15.44 (1.34)	15.05 (1.43)
Parent age	45.02 (6.07)	46.55 (5.49)
Parent education^a^	2.24 (1.36)	2.26 (1.35)
Adolescent gender, % female (n)	53 (148)	42 (187)
Parent relationship, % mothers (n)	89 (246)	74 (330)
Australian born, % (n^b^)	92 (187)	94 (301)

^a^Parent education is calculated as a score based on a 0-5 scale from year 10 school certificate to postgraduate education.

^b^n for the country of birth variable was 204 for the present study and 320 for the Hunter schools study.

## Discussion

### Comparative Effectiveness of Recruitment Methods

Facebook advertising was time-efficient and cost-effective for recruiting parents of 13-17 year olds to participate in our study. While specific costs were not attached to the traditional recruitment methods, the researcher time associated with this phase was substantial. In contrast, the development and dissemination of a Facebook advertisement were fast and straightforward. The cost per respondent AUD5.94 was lower than, or similar to, other studies reporting costs of US $20 [7], US $4.20 [5], and AUD12 [6] per completed survey or registered participant. (Note, in 2013, AUD1 was approximately equal to US $1).

The effect size would be over estimated if approach (1) continued to have effects during the implementation of approach (2), and/or that the combination of approaches (1) and (2) is greater than (2) alone. This is judged unlikely given the small effect size of (1) and the rapidity of the onset of a response after (2) was administered. While we did not utilize the “conversion tracking” feature of Facebook advertising, future studies could directly measure website visits resulting from a Facebook advertisement using this feature, which would be a more specific measure of advertisement success than the number of clicks.

Recruitment of large numbers of participants at low cost is a potentially powerful use of Facebook advertising, but the most appropriate target populations and research topics amenable to this type of recruitment should be considered. While Facebook is pervasive, variation exists by age in terms of member numbers and the extent of engagement.

### Different Types of Facebook Users

People 35-54 years old constitute a third of Facebook users [1], but based on user types defined by Evans et al [14], this does not equate to the proportion likely to participate in research. Older Facebook users are predominantly categorized as “neutrals” or “gamers”, accounting for 23% and 4% of Facebook users respectively, but with vastly different levels and types of use [14]. Neutrals have low engagement in terms of time spent on the site and frequency of visits, primarily using it to stay connected and informed about social events. Gamers are the smallest group numerically, but largest in terms of level of use. Evans et al [14] report that the primary motivation for gamers to engage in groups or link with others is the attainment of extrinsic rewards such as coupons and gaming points. Anecdotal evidence from members of the HPAF suggests that the majority of the parents who engaged in that part of the study were gamers. While neutrals may represent a large proportion of our prospective target population, attracting these users to engage in research and networking is challenging.

### Considerations for Recruiting Through Facebook

The study populations generated through Facebook are arguably more socially engaged, educated, and higher income groups than the general population. Several studies, however, report successfully recruiting participants that reflect the demographic spread reported in population surveys and census data [3,7]. In our study, the parents who completed the survey had diverse education levels and income, and reflected the population of Hunter schools in terms of demographics and ethnic diversity. In epidemiological analysis, diversity in the exposures of interest is more important than representativeness in estimating associations with outcomes [15].

An important consideration highlighted by our trial is the trade off between specificity and reach. Many potential participants may not have current or detailed profiles, such that they do not receive targeted advertising. More relaxed parameters increase the likelihood that an advertisement will be viewed by members of the target population, but decrease cost-effectiveness. It is possible that the pattern of response reflects the relationship between the relevance of an advertisement for an individual and their likelihood of responding. We found stricter parameters more economical in terms of the clicks/survey completions trade off.

The restrictive geographic inclusion criteria we used limited our participant numbers. Facebook recruitment approaches may be most appropriate for targeting large geographically and demographically diverse population groups, or for identifying highly targeted groups based on specific inclusion criteria associated with their profile or location.

## Abbreviations

HPAF: Hunter Parents Alcohol Forum
